# Integrative genomic SEM analysis of shared genetic architecture and candidate genes for insulin resistance–related metabolic traits

**DOI:** 10.1371/journal.pone.0361601

**Published:** 2026-10-29

**Authors:** ShuYan Li, Yi Liang, Guangwen Zhu, Lijiao Wu, Hong Li, Yu Li, Qiu Chen

**Affiliations:** 1 Faculty of Chinese Medicine, Macau University of Science and Technology, Macau, China; 2 Hospital of Chengdu University of Traditional Chinese Medicine, Chengdu, China; 3 Key Laboratory of Sichuan Province Ophthalmopathy Prevention & Cure and Visual Function Protection With TCM, Chengdu, China; 4 The First Affiliated Hospital of Chongqing University of Chinese Medicine, Chongqing, China; International Medical University, MALAYSIA

## Abstract

Insulin resistance is a complex metabolic state involving impaired insulin sensitivity, compensatory insulin secretion, abnormal lipid metabolism, and altered fat distribution. However, the shared genetic architecture underlying these correlated phenotypes remains incompletely defined. In this study, we applied Genomic Structural Equation Modeling to genome-wide association summary statistics of fasting insulin, insulin sensitivity index, triglycerides, and waist-to-hip ratio to construct an insulin resistance-related latent genetic factor. SNP-level association analysis was then performed for the latent factor, followed by risk locus annotation, fine-mapping, transcriptome-wide association analysis, gene-level prioritization, enrichment analysis, and exploratory structural biology assessment of selected missense substitutions. The single-factor model showed acceptable but not optimal approximate fit and captured a biologically consistent genetic pattern, with positive loadings for fasting insulin, triglycerides, and waist-to-hip ratio and a negative loading for insulin sensitivity index. The latent factor showed detectable SNP heritability and revealed 3,067 genome-wide significant candidate SNPs, with Q_SNP retained as a complementary SNP-level heterogeneity diagnostic.. Functional annotation identified 100 genomic risk loci and 1,530 FDR-significant genes in MAGMA. Fine-mapping prioritized multiple candidate regions, including loci near PPARG, RSPO3, SLC38A11, SLC39A8, BCL2, GDF5, and SPATA5. TWAS and FOCUS further highlighted candidate genes involved in adipocyte biology, glucose–lipid metabolism, insulin secretion, and expression regulation, including PPARG, MLXIPL, TCF7L2, FAM13A, CCDC92, and SLC22A3. Enrichment analyses suggested that genetic signals associated with the insulin resistance-related latent factor were mainly linked to fat distribution, cardiometabolic traits, adipose tissue, mesenchymal cell lineages, and active regulatory genomic regions. In a separate exploratory analysis, AlphaFold3 modeling, ThermoMPNN prediction, and molecular dynamics simulations suggested structural perturbations for three ultra-rare missense substitutions in nominated candidate proteins: BCL2-W188G, GDF5-C400Y, and RSPO3-C129Y. These substitutions were selected using functional-impact annotations and modeling feasibility, were not GWAS lead or fine-mapped causal variants, and do not establish the mechanisms underlying the GWAS associations. These findings provide an integrated genetic and structural framework for understanding shared mechanisms underlying insulin resistance-related metabolic abnormalities, while emphasizing the need for further experimental validation.

## 1. Introduction

Insulin resistance (IR) refers to a reduced responsiveness of target tissues to the biological actions of insulin [1,2]. It is characterized primarily by impaired glucose uptake in skeletal muscle, increased hepatic glucose production, dysregulated metabolic control in adipose tissue, and compensatory hyperinsulinemia [2,3]. Its development and progression are closely related to abnormal fat distribution, lipid metabolic disturbance, chronic low-grade inflammation, oxidative stress, and impaired insulin signaling [4,5]. As a common pathological basis of multiple metabolic disorders, insulin resistance is strongly associated with type 2 diabetes, obesity, metabolic syndrome, nonalcoholic fatty liver disease, and an increased risk of cardiovascular disease, often involving multiple organs and systems [2]. With the increasing prevalence of obesity, population aging, and lifestyle changes, the disease burden attributable to insulin resistance-related metabolic abnormalities has continued to rise, making insulin resistance a major challenge for cardiometabolic health [6]. Although progress has been made in the clinical assessment, mechanistic investigation, and therapeutic management of insulin resistance, its genetic basis and the biological mechanisms shared across related phenotypes remain incompletely understood [7,8]. Previous studies have suggested that adipose tissue dysfunction, reduced insulin sensitivity in skeletal muscle, abnormal hepatic glucose and lipid metabolism, and impaired vascular endothelial function may contribute to the onset and progression of insulin resistance [1,9]. However, these mechanisms do not fully explain interindividual differences in insulin sensitivity, metabolic phenotypes, or susceptibility to related diseases.

To address the limitation that a single clinical indicator cannot fully capture insulin resistance, this study applied Genomic Structural Equation Modeling (Genomic SEM) to integrate published GWAS summary statistics of insulin resistance-related phenotypes. Based on fasting insulin, the insulin sensitivity index, triglycerides, and waist-to-hip ratio, we constructed an insulin resistance-related latent factor to represent the shared metabolic genetic component underlying these phenotypes [7,10–12]. By modeling the shared genetic structure across different phenotypes, we further estimated the genetic associations between single-nucleotide polymorphisms (SNPs) and this latent factor. We combined genetic locus identification, candidate gene prioritization, tissue expression enrichment, and cell-type enrichment to characterize the shared genetic component. A separate computational structural analysis explored selected amino-acid substitutions in nominated candidate proteins.

It should be noted that this latent variable should not be regarded as a direct diagnostic indicator of insulin resistance. Rather, it is more appropriately interpreted as an insulin resistance-related shared metabolic genetic phenotype. In addition, this study focused on missense variants in core candidate genes that may affect protein structure and function. By integrating AlphaFold-predicted structures, confidence assessment of mutation sites, thermodynamic stability prediction, and molecular dynamics simulations, we explored the effects of representative variants on protein conformation, local stability, and dynamic properties. Overall, this study aimed to establish an analytical framework linking genetic statistical discovery to structural biological interpretation, thereby providing a theoretical basis for subsequent functional validation and the mechanistic investigation of prioritized candidate genes.

## 2. Methods

### 2.1 GWAS data sources and quality control

This study included four publicly available GWAS summary statistics closely related to insulin resistance as input phenotypes, including fasting insulin (FI), the insulin sensitivity index (ISI), triglycerides (TG), and waist-to-hip ratio (WHR). These phenotypes characterize insulin resistance-related metabolic status from the perspectives of compensatory insulin secretion, peripheral insulin sensitivity, lipid metabolic disturbance, and fat distribution, respectively. All input data were derived from published or publicly accessible GWAS summary statistics based on European-ancestry populations. The original studies had obtained the required ethical approval and informed consent from participants. Details on the sample sources, phenotype definitions, sample sizes, and download information for each dataset are provided in S1 Table.

Before multivariable genetic modeling, all input GWAS datasets were uniformly formatted and subjected to quality control. Variants located within the major histocompatibility complex region (MHC; chromosome 6: 25–35 Mb, GRCh37) were first removed to reduce the influence of the complex linkage disequilibrium structure in this region on genetic covariance estimation. Using the European reference panel from the 1000 Genomes Project Phase 3 as the LD reference, autosomal SNPs were then standardized and filtered [13]. Variants with a minor allele frequency (MAF) below 0.01, missing or zero effect estimates, inconsistent variant information relative to the reference panel, or allele orientations that could not be reliably harmonized were excluded. The quality-controlled summary statistics were used for subsequent genetic covariance estimation, Genomic SEM modeling, and SNP-level association analyses.

**Ethics statement.** This study analyzed publicly available GWAS summary statistics. Ethical approval and informed consent had been obtained in the source studies. No new individual-level data were collected or analyzed; therefore, additional ethical approval and consent for publication were not applicable.

### 2.2 Genomic structural equation modeling and latent factor GWAS

To identify the shared genetic structure across multiple insulin resistance-related phenotypes, this study constructed a genomic structural equation model using the GenomicSEM R package (version 0.0.5). This approach estimates genetic covariance among multiple complex phenotypes from GWAS summary statistics and identifies cross-phenotype shared genetic susceptibility components within a latent variable framework. It can also account for the potential influence of sample overlap and differences in sample size on model estimates [14].

The Genomic SEM analysis was performed in two steps. First, multivariable LD score regression was used to estimate the empirical genetic covariance matrix and the corresponding sampling covariance matrix among FI, ISI, TG, and WHR. Within the same LDSC framework, SNP-based heritability, the number of retained SNPs, genomic inflation factor (λGC), mean chi-square statistic, LDSC intercept, and attenuation ratio were extracted for each input GWAS dataset to evaluate polygenic signal and potential inflation of association statistics. Second, a single-factor structural equation model was fitted based on the genetic covariance matrix to evaluate whether the four metabolic phenotypes could be explained by a common insulin resistance-related latent genetic factor. Model fit was interpreted using pragmatic benchmarks of CFI ≥ 0.90 and SRMR ≤ 0.08 for acceptable approximate fit, with CFI around 0.95 treated as a stricter benchmark for stronger fit; the chi-square test was reported alongside these indices. Attenuation ratios were treated as quality-control diagnostics and were not interpreted quantitatively when the LDSC intercept was below 1. After fitting the model, SNP-level Genomic SEM GWAS was performed to estimate the association between each autosomal SNP and the latent genetic factor.

### 2.3 SNP heterogeneity test and statistical thresholds

Q_SNP was calculated as a complementary SNP-level diagnostic of departures from the effect pattern expected under the common-factor model. Non-significant Q_SNP values were not interpreted as formal evidence of effect homogeneity. Downstream analyses were conducted using the complete post-QC common-factor GWAS summary statistics, with Q_SNP retained to aid the interpretation of individual loci. For statistical significance, P < 5 × 10^−8^ was used as the genome-wide association threshold. More stringent thresholds of P < 5 × 10^−12^ and P < 5 × 10^−16^ were used for descriptive stratification of association strength.

### 2.4 Risk locus definition, functional annotation, and novel locus identification

Based on the Genomic SEM GWAS summary statistics, this study used the FUMA platform to define genomic risk loci and perform functional annotation [15]. Independent significant SNPs reaching genome-wide significance and showing low linkage disequilibrium with one another were first identified, and lead SNPs were then determined accordingly. SNP independence was defined using an r² threshold of < 0.1. Adjacent significant signals were subsequently merged into risk loci according to physical distance and local LD clustering.

To determine whether the multivariable model identified genetic signals not captured by single-phenotype GWAS, lead SNPs detected by Genomic SEM were systematically compared with known association loci reaching the same significance threshold in the univariate GWAS of FI, ISI, TG, and WHR. A lead SNP was defined as a potentially novel locus if it was located more than 1 Mb away from any significant locus reported in the corresponding univariate GWAS. In addition, a “GWAS-minus-locus” comparison strategy was applied, in which Genomic SEM lead loci were sequentially compared against significant loci from the four input GWAS in an exclusion-based manner to identify independent candidate variants that emerged only in the shared genetic structure [16].

To evaluate the potential pleiotropic features of the lead loci, this study further queried the GWAS Catalog for genome-wide significant associations between lead SNPs and other published complex traits [17]. Based on the risk loci defined by FUMA, MAGMA was used to perform gene-level association analysis [18]. By integrating the genetic effects of multiple SNPs within gene regions, MAGMA estimated the overall association strength between candidate genes and the insulin resistance-related latent factor. Genes with an FDR < 0.05 were defined as candidate genes. Genes surviving Bonferroni correction were further defined as high-confidence genes and used as the input for the main gene-set enrichment analysis.

### 2.5 Fine-mapping analysis

To narrow the candidate set of potential causal variants, this study performed statistical fine-mapping analyses for the major risk regions identified by Genomic SEM GWAS. For each lead SNP, local association variants within a ± 250-kb window were extracted. Using LD reference data from the European population in the 1000 Genomes Project Phase 3, two Bayesian fine-mapping methods, SuSiE and FINEMAP, were applied separately to estimate the posterior inclusion probability (PIP) within each SNP within the region [19,20]. Both methods were used to construct 95% credible sets, and the resulting candidate variants were cross-validated between methods. Variants supported by both SuSiE and FINEMAP and showing relatively high posterior inclusion probabilities were prioritized for locus-level interpretation. Candidate genes for structural exploration were nominated from prioritized loci using locus-to-gene annotation. The missense substitutions subsequently retrieved within these genes were selected separately and were neither GWAS lead variants nor variants prioritized as putatively causal by these fine-mapping analyses. The fine-mapping evidence at these loci therefore does not constitute variant-level genetic evidence for the modeled substitutions.

### 2.6 Transcriptome integration analysis and candidate gene prioritization

To identify candidate genes that may contribute to insulin resistance-related metabolic abnormalities through gene expression regulation, this study performed a transcriptome-wide association study (TWAS) based on the Genomic SEM GWAS results. TWAS was conducted using FUSION, with GTEx v8 cross-tissue sCCA expression weight models used as the reference [21–23]. Multiple testing was controlled by Bonferroni correction across 37,917 gene–panel models, giving a significance threshold of 1.3187 × 10^−6^ (0.05/37,917). By integrating GWAS association statistics with expression quantitative trait locus information, this analysis evaluated the association between genetically predicted gene expression levels and the insulin resistance-related latent genetic factor.

For statistically significant TWAS signals, FOCUS was further applied for Bayesian gene-level fine-mapping to calculate the posterior inclusion probability (PIP) of candidate genes and construct credible gene sets [24]. Finally, high-confidence candidate functional genes were prioritized by integrating TWAS significance, FOCUS prioritization, FUMA functional mapping, and MAGMA gene-level association results.

### 2.7 Gene-set, disease ontology, and phenotype ontology enrichment

To interpret the genetic association results at the pathway and disease annotation levels, this study performed gene-set and pathway enrichment analyses using MAGMA and FUMA. These analyses were used to evaluate whether the candidate genes showed aggregation in specific biological processes or functional pathways. MendelVar was further used to perform Disease Ontology and Human Phenotype Ontology enrichment analyses for candidate genes related to significant risk regions, in order to assess whether these shared genetic signals were preferentially enriched in disease and phenotype categories related to metabolic abnormalities, fat distribution, cardiovascular changes, inflammatory responses, or endocrine regulation [25].

### 2.8 Cell-type-specific enrichment analysis

To explore the cellular sources through which shared genetic signals for insulin resistance may act, this study performed cell-type-specific enrichment analysis using the CELLECT framework [26]. First, based on the Tabula Muris single-cell RNA sequencing reference dataset, CELLEX was used to calculate expression specificity scores for each gene across different cell types [27]. These scores were then integrated with the Genomic SEM GWAS summary statistics, and stratified LD score regression was applied to evaluate the degree of heritability enrichment of genetic variants in cell-type-specific expressed gene sets [28]. Multiple testing was controlled using FDR correction, with an FDR-adjusted P < 0.05 defined as significant cell-type enrichment. Nominally significant results that did not survive FDR correction were interpreted only as suggestive findings.

### 2.9 Partitioned heritability analysis

Beyond tissue and cell-type annotations, this study further applied partitioned LD score regression to evaluate the contribution of different functional genomic annotations to the SNP heritability of the insulin resistance-related latent factor [29]. This analysis integrated functional annotations, linkage disequilibrium structure, and GWAS association statistics to estimate heritability enrichment across different categories of functional genomic regions. By comparing heritability enrichment across annotation categories, we assessed whether the shared genetic signals were preferentially concentrated in genomic regions with regulatory activity or protein-coding function.

### 2.10 Summary statistics-based polygenic risk score analysis

To evaluate the polygenic genetic predisposition of the insulin resistance-related latent factor from the perspective of overall genetic burden, this study constructed polygenic risk scores (PRSs) based on the Genomic SEM GWAS summary statistics. PRS analysis was performed using PRS-CS, which estimates posterior SNP effect sizes across the genome through a Bayesian continuous shrinkage prior model combined with an external European LD reference panel [30]. Genome-wide PRSs were then calculated based on all available autosomal variants. To further compare the relative genetic contribution of different chromosomal regions, chromosome-stratified PRSs were also constructed, and the association between chromosome-specific scores and the genetic risk represented by the latent factor was evaluated.

### 2.11 Missense variant selection and protein structure preparation

Variant selection followed two separate stages. First, GWAS-associated loci and statistical fine-mapping results, together with FUMA locus-to-gene annotation and integrated genetic evidence, informed candidate-gene nomination. Second, missense substitutions in the nominated genes BCL2, GDF5, and RSPO3 were retrieved from Ensembl GRCh38 records. Candidate substitutions were reviewed using available functional prediction annotations, including SIFT, PolyPhen-2, CADD, REVEL, MetaLR, and Mutation Assessor, together with available clinical annotations, AlphaFold confidence at the affected residues, amino-acid substitution type, protein size, structural interpretability, and feasibility of comparative structural modelling and molecular-dynamics simulation. One substitution per gene was selected qualitatively for exploratory structural analysis; no formal pathogenicity-ranking algorithm was applied. Within the nominated genes, selection was based on predicted functional impact and modelling suitability, not on variant-level GWAS association strength or fine-mapping posterior probability. Computational pathogenicity predictions were distinguished from clinical classifications, and neither was interpreted as evidence of association with the IR-related latent factor. Population frequency was not used in the original selection. During revision, population frequencies were checked in the gnomAD v4.1.1 browser using GRCh38 coordinates and the reference-to-alternate allele for each modeled substitution. The combined exome/genome allele frequency, where available, is reported in Table 2. A browser result of “Variant not found” is reported as “Not detected” rather than assigned a numerical frequency of zero. The selected substitutions are ultra-rare, being absent or observed only as a singleton in this gnomAD release. They were neither GWAS lead variants nor fine-mapped causal variants. This step was intended to generate protein-structure hypotheses and was not a functional validation of the common-variant GWAS signals. The reference protein sequences, transcript information, and amino acid numbering of candidate proteins were cross-checked using Ensembl, UniProt, and NCBI Gene to ensure consistency between genetic variants and protein sequence positions [31].

Wild-type amino acid sequences were then obtained, and corresponding mutant sequences were generated according to the missense variant information. For proteins lacking complete high-resolution experimental structures, AlphaFold3-predicted structures were used as the basis for subsequent simulations [32]. The pLDDT score was used to assess the structural confidence of the mutation sites and adjacent regions. Both wild-type and mutant structures were inspected in PyMOL to confirm the integrity of structure files, the localization of mutation sites, and the plausibility of side-chain conformations [33].

### 2.12 Molecular dynamics simulation and trajectory analysis

To evaluate the effects of missense variants on global conformational stability and local dynamic behavior of proteins, this study performed molecular dynamics simulations using GROMACS 2024.5 [34]. System construction was carried out with the AMBER14SB force field and the TIP3P explicit water model. Each protein was placed in a cubic water box with a minimum distance of 1.0 nm from the box boundary. Na+ and Cl− ions were added to neutralize the system charge, and 0.15 M NaCl was introduced to mimic physiological ionic strength. All systems were first subjected to energy minimization using the steepest descent algorithm to remove unfavorable atomic contacts. This was followed by 100 ps NVT equilibration and 100 ps NPT equilibration to stabilize the systems at 300 K and 1 atm. After equilibration, 100 ns production simulations were performed for each wild-type protein and its corresponding mutant. The integration time step was set to 2 fs, and all systems were simulated under identical parameter settings to ensure consistency in between-group comparisons.

Simulation trajectories were used for multidimensional analyses of structural stability. Root mean square deviation (RMSD) was first calculated to assess global conformational drift. Root mean square fluctuation (RMSF) was then calculated to compare residue-level changes in flexibility. Radius of gyration (Rg), solvent accessible surface area (SASA), and the number of intramolecular hydrogen bonds were also analyzed to comprehensively evaluate protein compactness, solvent exposure, and internal stability. For regions adjacent to mutation sites, trajectory visualization was further used to examine changes in local secondary structure, side-chain orientation, and interaction networks. These simulation results were used mainly to compare relative dynamic differences between wild-type and mutant proteins and should not be directly interpreted as absolute functional states in the complex in vivo microenvironment.

### 2.13 Mutation-induced thermal stability prediction

In addition to molecular dynamics trajectory analysis, this study further used ThermoMPNN to predict the mutation-induced change in free energy (ΔΔG) caused by missense variants [35]. The three-dimensional structure of the wild-type protein and the corresponding mutation-site information were used as input, and the official pretrained model was applied to calculate the predicted ΔΔG value for each missense variant. In general, a positive ΔΔG suggests that a mutation may reduce protein thermodynamic stability, whereas a negative or near-zero value indicates a relatively limited effect on structural stability. The ThermoMPNN predictions were then compared with the trends observed for RMSD, RMSF, Rg, SASA, and hydrogen-bond changes. This integrated analysis assessed the potential structural and functional effects of representative missense variants from the complementary perspectives of static thermal stability and dynamic conformational behavior.

## 3. Results

### 3.1 Genomic SEM latent factor construction and model fit

Based on four insulin resistance-related metabolic phenotypes, including fasting insulin (FI), the insulin sensitivity index (ISI), triglycerides (TG), and waist-to-hip ratio (WHR), we first estimated the genetic covariance matrix using multivariable LD score regression. LDSC showed that all four input GWAS datasets had detectable SNP heritability. The observed-scale SNP heritability estimates were h² = 0.0815 for FI (SE = 0.0121), h² = 0.1042 for ISI (SE = 0.0117), h² = 0.1478 for TG (SE = 0.0160), and h² = 0.1352 for WHR (SE = 0.0084), with corresponding h² Z values of 6.72, 8.88, 9.26, and 16.0, respectively. Detailed LDSC quality-control metrics, including the number of retained SNPs, λGC, mean chi-square statistics, LDSC intercepts, and attenuation ratios, are summarized in Table 1. Pairwise genetic correlation analysis showed FI–ISI = −0.8392, ISI–TG = −0.3319, and ISI–WHR = −0.4424, whereas FI–TG = 0.3654, FI–WHR = 0.4571, and TG–WHR = 0.3469. The complete genetic correlation matrix is shown in S2 Table and Fig 1.

**Table 1:** SNP-based heritability and LD score regression quality control for the four metabolic phenotypes.

Phenotype	N SNPs	SNP-based h² (SE)	λGC	Mean χ²	LDSC intercept (SE)	Attenuation ratio (SE)
FI	1,108,409	0.0815 (0.0121)	1.0708	1.0900	1.0077 (0.0071)	0.0856 (0.0793)
ISI	1,168,587	0.1042 (0.0117)	1.0465	1.0705	0.9609 (0.0072)	−0.5543 (0.1018)
TG	1,163,413	0.1478 (0.0160)	1.2544	1.3891	1.0357 (0.0174)	0.0918 (0.0447)
WHR	1,180,866	0.1352 (0.0084)	1.5666	2.0024	1.0933 (0.0216)	0.0931 (0.0216)

Abbreviations: FI, fasting insulin; ISI, insulin sensitivity index; TG, triglycerides; WHR, waist-to-hip ratio; SNP, single-nucleotide polymorphism; LDSC, LD score regression; SE, standard error; λGC, genomic inflation factor. SNP-based h² estimates were calculated using univariate LD score regression. Attenuation ratios were treated as quality-control diagnostics; when the LDSC intercept was below 1, they were not interpreted quantitatively.

**Fig 1:**
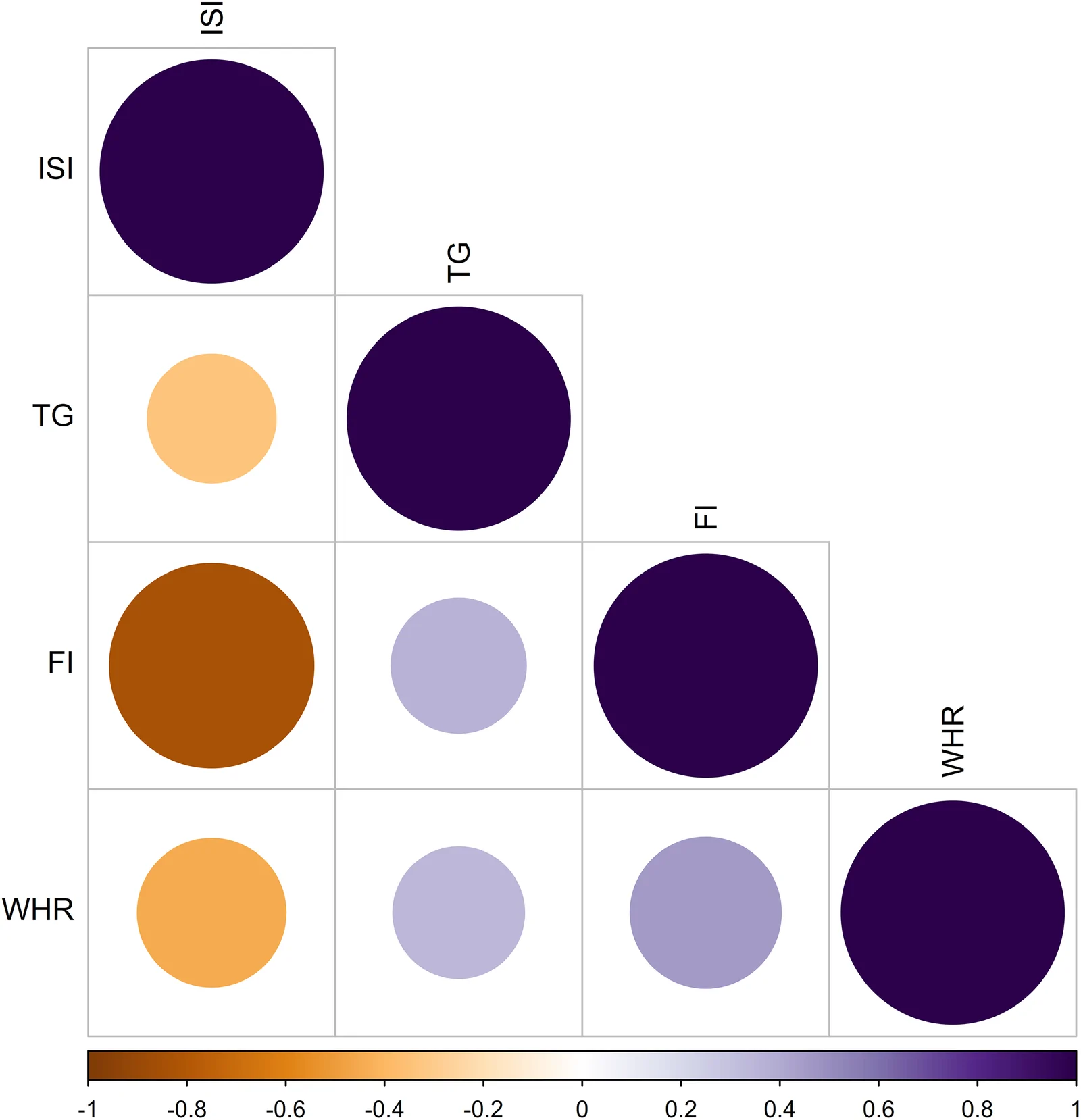
Genetic correlation matrix among insulin resistance-related metabolic phenotypes. Circle size represents the absolute magnitude of the genetic correlation, whereas color represents its signed direction and magnitude. Orange indicates negative genetic correlation and purple indicates positive genetic correlation, with the color scale ranging from −1 to 1.

Based on the genetic covariance matrix, we further constructed a single-factor Genomic SEM model. The model fit indices were χ² = 23.486, df = 2, P = 7.94 × 10^−6^, AIC = 39.486, CFI = 0.9399, and SRMR = 0.0494, indicating acceptable but not optimal approximate fit under the pragmatic criteria of CFI ≥ 0.90 and SRMR ≤ 0.08; CFI around 0.95 was treated as a stricter benchmark. Standardized factor loadings showed that FI, TG, and WHR were positively associated with the latent factor, with loadings of 0.924, 0.467, and 0.557, respectively. ISI was negatively associated with the latent factor, with a standardized loading of −0.822. The factor was oriented so that FI had a positive loading. Under this convention, TG and WHR also loaded positively, whereas ISI loaded negatively. The direction of these loadings was consistent with the biological pattern of insulin resistance, in which higher fasting insulin, triglyceride levels, and waist-to-hip ratio, together with lower insulin sensitivity, indicate a greater IR-related shared metabolic genetic tendency. Model stability assessment is presented in S3a Table, and the path parameters between latent factor F1 and the input phenotypes are shown in S3b Table. The exploratory factor model is provided in S1 File. This latent factor reflects the shared genetic component across insulin resistance-related metabolic phenotypes rather than a single clinical diagnostic measure of IR.

### 3.2 SNP-level genomic SEM GWAS and genomic control

Based on the common factor model, we further performed SNP-level Genomic SEM GWAS. The original summary statistics contained 2,374,423 SNPs. After LDSC formatting, 1,295,874 SNPs were removed because they were not included in the HapMap3 SNP reference list, leaving 1,078,549 valid SNPs. After merging with the European LD reference panel, 1,077,894 SNPs were included in heritability estimation and subsequent analyses.

Genomic control results showed that the mean χ² statistic of the latent factor was 1.6344, with a lambda GC of 1.4034 and a maximum χ² statistic of 287.618. The LDSC intercept was 0.9755 (SE = 0.0139), close to 1 and not suggestive of substantial residual inflation attributable to confounding. Because the intercept was below 1, the corresponding negative attenuation ratio was not interpreted quantitatively.. The observed-scale SNP heritability was h² = 0.0599 (SE = 0.0028), indicating a detectable polygenic basis for the latent factor.

Using P < 5 × 10^−8^ as the genome-wide association threshold, we identified 3,067 candidate SNPs. More stringent thresholds identified 501 SNPs at P < 5 × 10^−12^ and nine SNP-level signals at P < 5 × 10^−16^. Q_SNP was examined separately as a complementary SNP-level diagnostic. The complete post-QC common-factor summary statistics were retained as inputs for FUMA/MAGMA, fine-mapping, TWAS/FOCUS, cell-type enrichment, partitioned LDSC, and PRS analyses. Among them, rs998584, rs6905288, and rs1358980 were located in the 6p21.1 VEGFA/POLR1C-related region, and rs17144687 was located near DTWD2. The complete results are provided in the Science Data Bank repository (https://www.scidb.cn/anonymous/ZW1NQlZ6), S4a and S4b Table.

### 3.3 Lead SNPs, risk loci, and gene-level results

To further summarize independent genetic signals, we used FUMA to perform functional annotation and genomic risk locus definition based on the Genomic SEM GWAS results. FUMA identified 100 genomic risk loci after merging adjacent association signals according to LD relationships and physical distance (S5 Table). At the gene level, MAGMA identified 1,530 candidate genes after FDR correction (FDR < 0.05). These genes were retained as the input gene set for subsequent gene-set enrichment analysis and functional interpretation (S6 Table and Fig 2). Functional annotation further identified 152 lead SNPs, most of which were located in non-coding regions, including intronic regions, non-coding RNA introns, and intergenic regions, suggesting that the associated genetic signals may act predominantly through regulatory variants (S7 Table). Through an exclusion-based comparison with significant loci from the univariate GWAS of FI, ISI, TG, and WHR, we further identified 142 candidate SNPs that may be driven primarily by the shared genetic structure (S8 Table).

**Fig 2:**
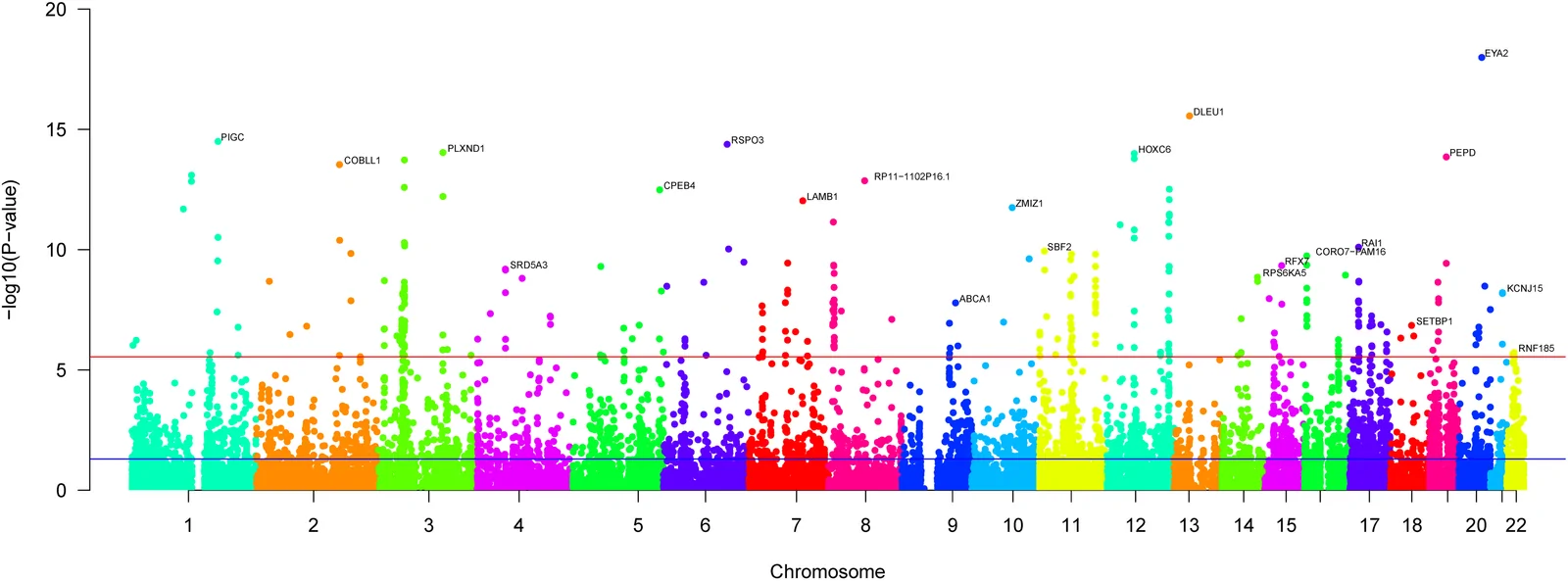
MAGMA gene-level association analysis of the insulin resistance-related latent genetic factor. Manhattan plot showing gene-level associations identified by MAGMA based on the Genomic SEM GWAS of the insulin resistance-related latent factor. Genes are arranged by chromosomal position, and the y-axis represents −log10(P-value). Horizontal reference lines indicate preset significance thresholds. Representative significant genes are annotated.

### 3.4 Fine-mapping analysis

To further narrow the set of putative causal variants, this study performed SuSiE and FINEMAP fine-mapping analyses for the major risk regions. The results showed that several regions contained prioritized candidate variants with relatively high posterior inclusion probabilities (PIPs), with consistent or comparable support across the two methods. Representative loci included rs4573145 near AC006458.3 in the 7q region, rs7220893 within DNAH9 in the 17p region, rs2249407 within PPARG in the 3p region, rs1128249 near RNA5SP111 in the 2q region, rs2745359 near RSPO3 in the 6q region, rs3820981 within SLC38A11 in the 2q region, and rs1813006 near SLC39A8 in the 4q region. In addition, regions near BCL2, BCL2L11, MECOM, GDF5, MAFB, SPATA5, and HOXC6/MIR196A2 showed varying degrees of posterior probability support. Regional association plots revealed clear association peaks at these loci (Fig 3 and S9 Table).

**Fig 3:**
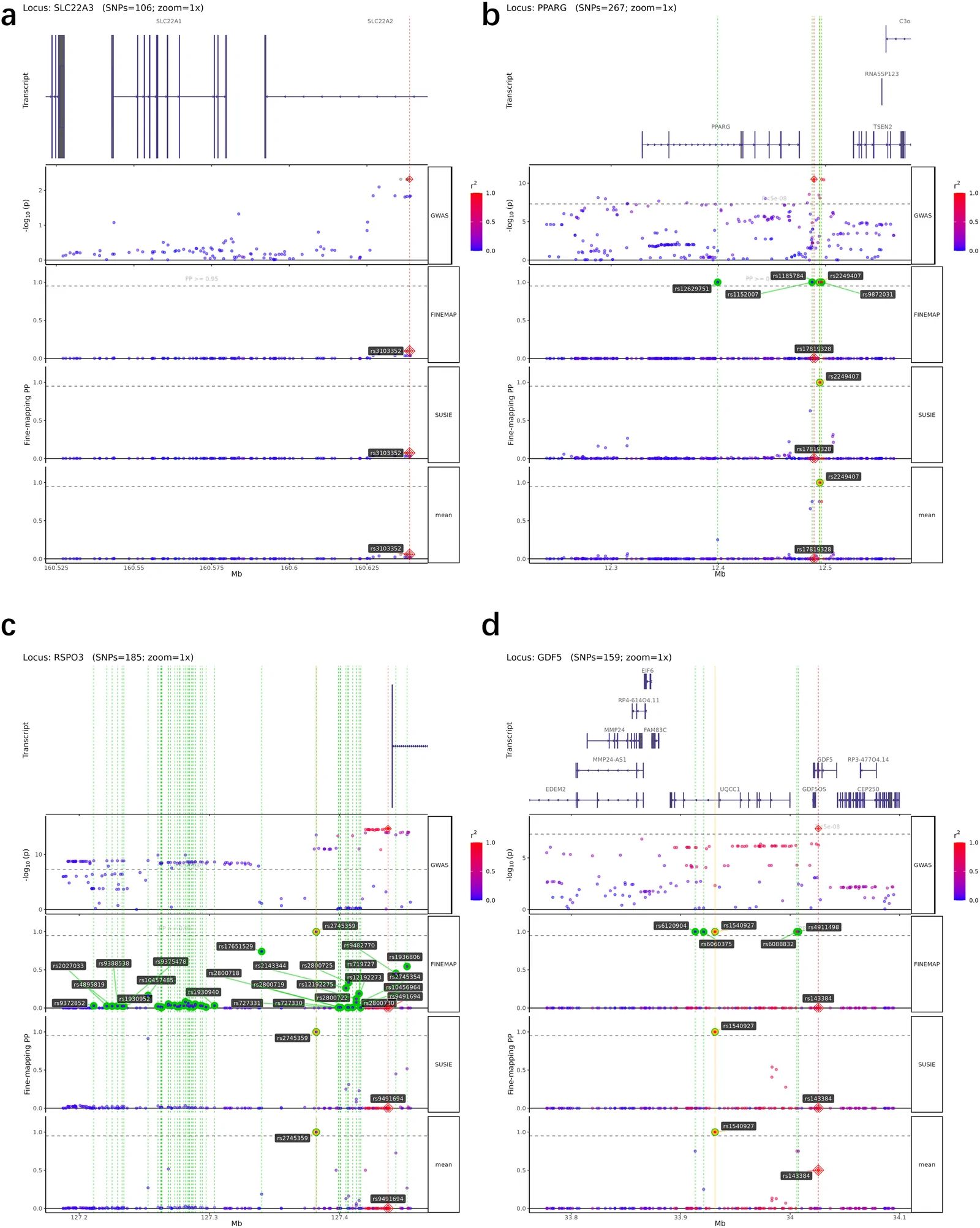
Regional association and fine-mapping plots of representative risk loci. Regional plots showing GWAS association strength and fine-mapping posterior inclusion probabilities for selected candidate loci. Panels show: **(a) SLC22A3**, **(b) PPARG**, **(c) RSPO3**, and **(d) GDF5**. For each locus, the upper panel displays regional association signals, while the lower tracks summarize FINEMAP, SuSiE, and mean fine-mapping posterior probabilities. Highlighted variants indicate prioritized candidate signals within each region.

### 3.5 TWAS/FOCUS candidate gene prioritization

This study further performed TWAS analysis using FUSION to evaluate the association between genetically predicted gene expression and the latent IR factor based on the GTEx v8 cross-tissue sCCA expression weight models. FUSION TWAS identified 402 significant gene–tissue association signals after Bonferroni correction across 37,917 gene–panel models. The detailed results are provided in S10 Table and Fig 4. Based on the significant TWAS results, FOCUS was further applied for Bayesian fine-mapping. Using TWAS multiple-testing significance and inclusion in FOCUS credible sets as joint filtering criteria, we identified 221 overlapping gene–panel-level signals, involving 147 unique candidate genes. Representative genes included CCDC92, BHLHE41, VEGFB, SNX10, MLXIPL, JAZF1, FAM13A, TCF7L2, PPARG, and SLC22A3.

**Fig 4:**
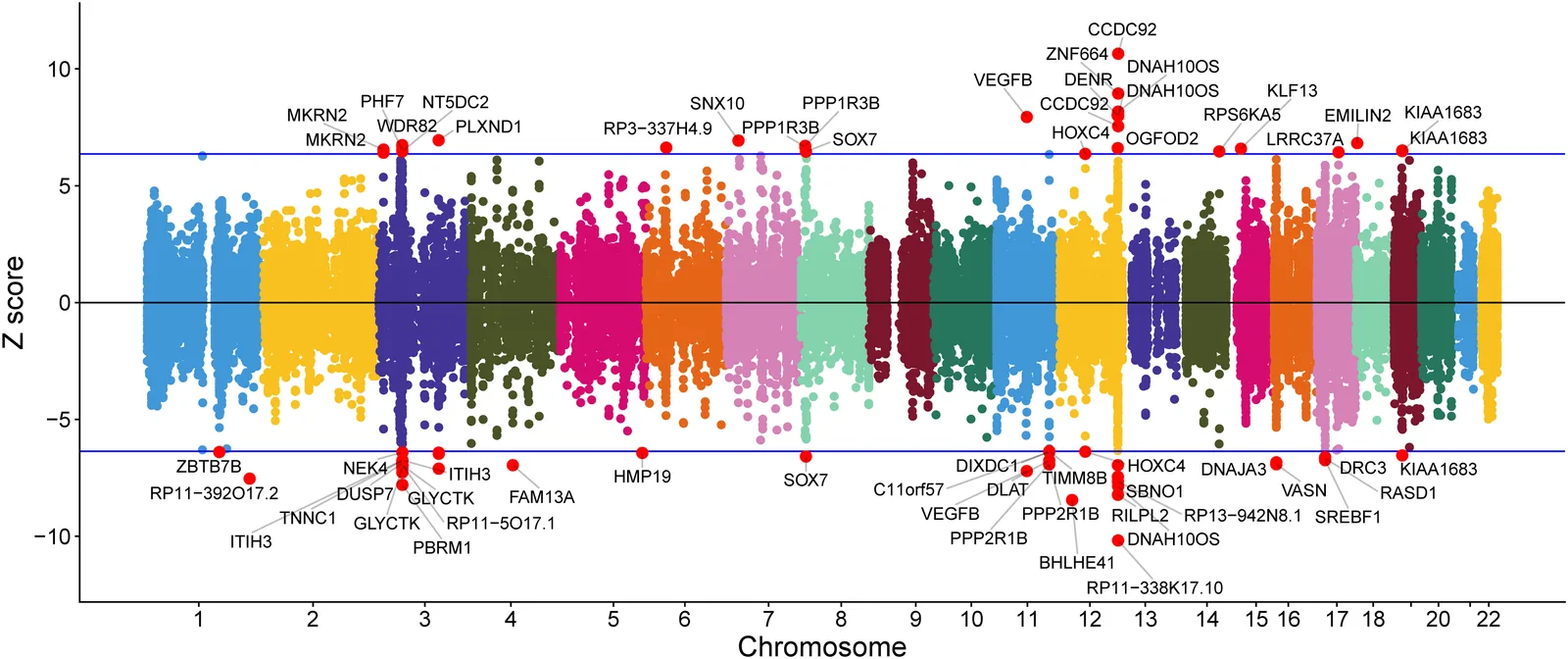
TWAS association landscape for the insulin resistance-related latent genetic factor. Manhattan plot of transcriptome-wide association study results based on FUSION using GTEx v8 cross-tissue sCCA expression weights. Genes are arranged by chromosomal position, and the y-axis represents TWAS Z scores. Positive and negative Z scores indicate opposite directions of association between genetically predicted gene expression and the latent factor. Genes exceeding Bonferroni-corrected significance threshold (P < 1.3187 × 10^−6^) are highlighted and annotated.

Directionality analysis showed that the genetically predicted expression of CCDC92, SNX10, MLXIPL, JAZF1, and PPARG was positively associated with the latent IR factor, whereas BHLHE41, FAM13A, and TCF7L2 showed negative associations. SLC22A3 and VEGFB showed inconsistent directions across different sCCA panels and therefore were not assigned a single directional interpretation. The overlapping TWAS and FOCUS results are presented in S11 Table.

### 3.6 Pathway, cell-type, and Mendelian disease gene enrichment

Based on the 317 genes that remained significant after Bonferroni correction in the MAGMA analysis(S12 Table), we further performed gene-set, pathway, and phenotype ontology enrichment analyses. The results showed that IR-related candidate genes were mainly enriched for fat distribution-related traits, including waist-to-hip ratio, BMI-adjusted waist-to-hip ratio, waist circumference, hip circumference, and body shape index. Enrichment was also observed for cardiometabolic phenotypes, including triglycerides, HDL cholesterol, fasting insulin, type 2 diabetes, metabolic syndrome, blood pressure, and coronary artery disease, suggesting that the genetic signals captured by the latent factor were closely related to fat distribution, glucose and lipid metabolism, and cardiometabolic regulation (S13 Table).

GO and GO-slim analyses further indicated that the candidate genes were mainly enriched in carbohydrate metabolism, protein glycosylation, receptor ligand activity, vesicle-mediated transport, endosomal and Golgi transport, endoplasmic reticulum function, and chromatin-related processes. Some terms were also related to responses to oxygen levels, cytoskeletal regulation, microtubule organization, mitosis, and DNA-templated transcription. Positional gene-set analysis showed that regions such as chr3p21, chr8p23, chr17p11, and chr12q24 may have contributed substantially to the candidate gene enrichment signals (Fig 5).

**Fig 5:**
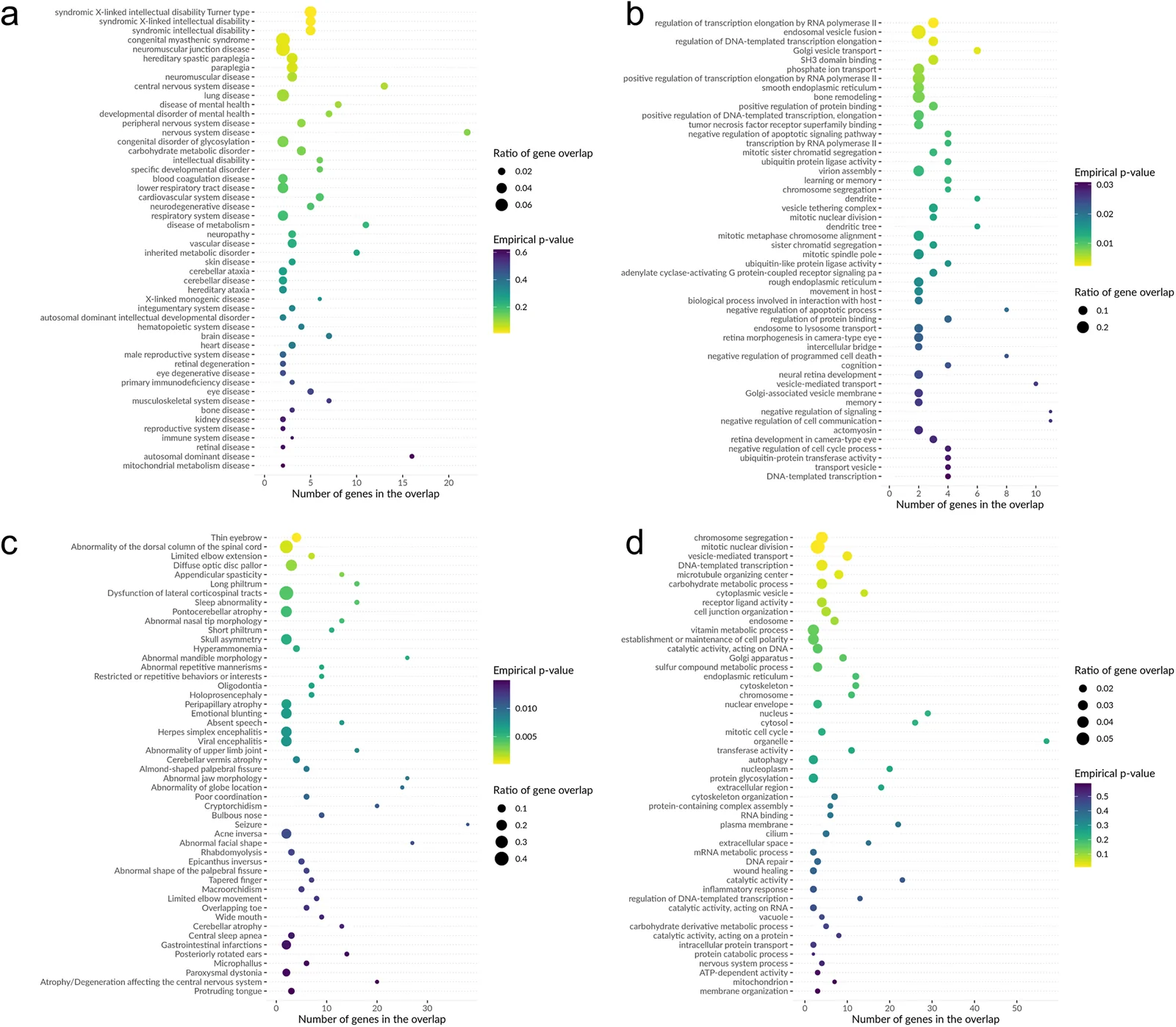
Functional, disease, and phenotype enrichment analyses of candidate genes. Bubble plots showing enrichment results for candidate genes associated with the insulin resistance-related latent factor. Panels show: (a) Disease Ontology enrichment, (b) Gene Ontology biological process enrichment, (c) Human Phenotype Ontology enrichment, and (d) GO-slim functional enrichment. The x-axis represents the number of overlapping genes. Dot size indicates the ratio of gene overlap, and dot color represents the empirical P-value.

Cell and tissue enrichment analyses showed that IR-related genetic signals mainly pointed to adipose tissue and mesenchymal cell lineages. In the single-cell analysis, limb muscle mesenchymal stem cells, tracheal mesenchymal cells, and adipose-derived mesenchymal stem cells survived FDR correction. Multi-tissue expression analysis and GTEx results also showed significant enrichment signals in subcutaneous adipose tissue and visceral adipose tissue. Chromatin annotation further showed significant enrichment of active regulatory marks, including H3K4me1, H3K9ac, and H3K27ac, in adipose nuclei, suggesting that some IR-related variants may be located in chromatin regions with regulatory activity in adipose tissue. Overall, these findings support adipose tissue and its associated stromal or mesenchymal cellular environment as a potential biological context for shared IR genetic signals. Results related to arteries, liver, and immune cells did not reach stable significance after correction and were therefore interpreted only as exploratory findings (S14a–S14d Table). MendelVar annotation enrichment analysis did not identify any Disease Ontology or Human Phenotype Ontology terms that survived FDR correction (FDR > 0.05; S15 and S16 Tables). Therefore, the top-ranked nominal signals in DO and HPO were used only as phenotypic annotation references for candidate genes and were not interpreted as significant enrichment results.

### 3.7 Heritability contribution across genomic functional regions

Partitioned LDSC analysis showed that the SNP heritability of the latent IR factor was mainly enriched in functionally active genomic regions, including conserved elements, DHS/DGF open chromatin regions, enhancers, super-enhancers, transcription factor binding sites, and regions near transcription start sites. Active regulatory marks, including H3K27ac, H3K4me1, H3K4me3, and H3K9ac, also showed significant enrichment. Among gene-proximal annotations, coding regions, extended coding regions, introns, extended promoter regions, and UTR regions showed varying degrees of enrichment. In contrast, repressed chromatin annotations had enrichment values below 1, suggesting that the associated genetic signals were less frequently located in transcriptionally repressed regions. These results indicate that the genetic contribution to IR-related variation mainly arises from open, conserved, and regulatory-active chromatin environments (S17 Table).

### 3.8 Chromosome-stratified distribution of genetic signals

Chromosome-stratified summary PRS analysis showed that IR-related genetic signals were unevenly distributed across chromosomes. Chromosomes 19, 22, 16, 12, and 4 showed major positive contributions, with chromosome 19 showing the largest contribution. In contrast, chromosomes 5, 18, 13, 20, 9, and 6 showed negative contributions. These findings suggest that the direction and magnitude of chromosomal contributions to the IR polygenic risk score differ across the genome. However, chromosome-level results reflect only aggregate genetic trends and should not be directly interpreted as specific causal loci or functional genomic regions (S18 Table).

### 3.9 Selection of representative missense substitutions for structural analysis

In the SuSiE and FINEMAP fine-mapping analyses, several risk regions received consistent or comparable support, involving candidate genes or nearby regions such as BCL2, BCL2L11, GDF5, PPARG, RSPO3, SLC38A11, SLC39A8, and SPATA5. Fine-mapping was used to prioritize candidate variants and associated regions, while candidate genes were nominated based on locus annotation and integrated genetic evidence. For the subsequent exploratory structural analysis, missense substitutions in BCL2, GDF5, and RSPO3 were reviewed using functional prediction annotations and structural-modelling criteria. One representative substitution per gene—BCL2-W188G, GDF5-C400Y, and RSPO3-C129Y—was taken forward for AlphaFold3 modelling, thermal-stability prediction, and molecular-dynamics simulation. These ultra-rare substitutions were selected for exploratory structural analysis and were neither GWAS lead variants nor fine-mapped causal variants. The Ensembl annotations reported CADD scores of 32, 31, and 32 and REVEL scores of 0.756, 0.986, and 0.816 for BCL2-W188G, GDF5-C400Y, and RSPO3-C129Y, respectively. These predictions supported functional-interest selection but did not provide evidence of association with the IR-related latent factor. In gnomAD v4.1.1, BCL2-W188G and GDF5-C400Y were not detected in the browser. RSPO3-C129Y was observed as a singleton, with a combined allele count of 1, allele number of 1,612,204, and allele frequency of 6.203 × 10^−7^ (Table 2). The single observation occurred in the exome dataset and passed its variant quality filter; no variant was observed in the genome dataset. Detailed functional annotations, transcript-specific predictions, and population frequencies of the three selected missense variants are provided in S19 Table.

**Table 2:** Candidate missense variants prioritized for protein structural analysis.

Gene	rsID	Position	Alternate Allele	Reference Allele	Protein change	gnomAD AF
GDF5	rs74315387	20:35434216	T	C	p.Cys400Tyr	Not detected
BCL2	rs2144321090	18:63318105	C	A	p.Trp188Gly	Not detected
RSPO3	rs1352673048	6:127150522	A	G	p.Cys129Tyr	6.203 × 10^−7^

Abbreviations: AF, allele frequency; REF, reference allele; ALT, alternate allele; rsID, reference SNP identifier.

To further clarify the genetic and protein-level localization information of these candidate missense variants, we systematically summarized the three genes and their major variant sites, as shown in Table 2. It should be noted that genes such as PPARG, MLXIPL, TCF7L2, and SLC39A8 remain important genetic or expreSssion-regulatory candidate genes. However, their supporting evidence was mainly derived from risk loci, pathway enrichment, or TWAS/FOCUS analyses, or their inclusion in molecular dynamics analysis was limited by protein structural complexity, prediction confidence, and interpretability of simulation results. Therefore, these genes were not included in the MD analysis in this study.

Positions and REF/ALT alleles refer to the GRCh38 reference genome in forward genomic orientation. Protein changes use HGVS-style three-letter amino-acid notation. Population frequencies are from the gnomAD v4.1.1 browser. The RSPO3 value is the combined exome/genome (Total) AF: allele count (AC) = 1 and allele number (AN) = 1,612,204. The observation occurred in the exome dataset (AC = 1; AN = 1,460,198; AF = 6.848 × 10^−7^; filter = Pass). The genome dataset reported no variant (AC = 0; AN = 152,006).Not detected denotes a “Variant not found” result for the specified GRCh38 allele in the gnomAD v4.1.1 browser. No numerical AF or site-specific AN was available in those variant results; a frequency of zero has not been assigned. These ultra-rare substitutions were selected within nominated candidate genes using functional-impact annotations and structural-modelling suitability. They were neither GWAS lead variants nor fine-mapped causal variants. Their structural analysis does not establish that they explain the common-variant GWAS associations.

### 3.10 Structural modeling analysis

AlphaFold3 prediction showed that the mutation sites of BCL2, GDF5, and RSPO3 and their adjacent regions were within an acceptable to relatively high pLDDT confidence range, suggesting that these predicted structures were suitable for subsequent comparative structural assessment and exploratory molecular dynamics simulations (Fig 6).

**Fig 6:**
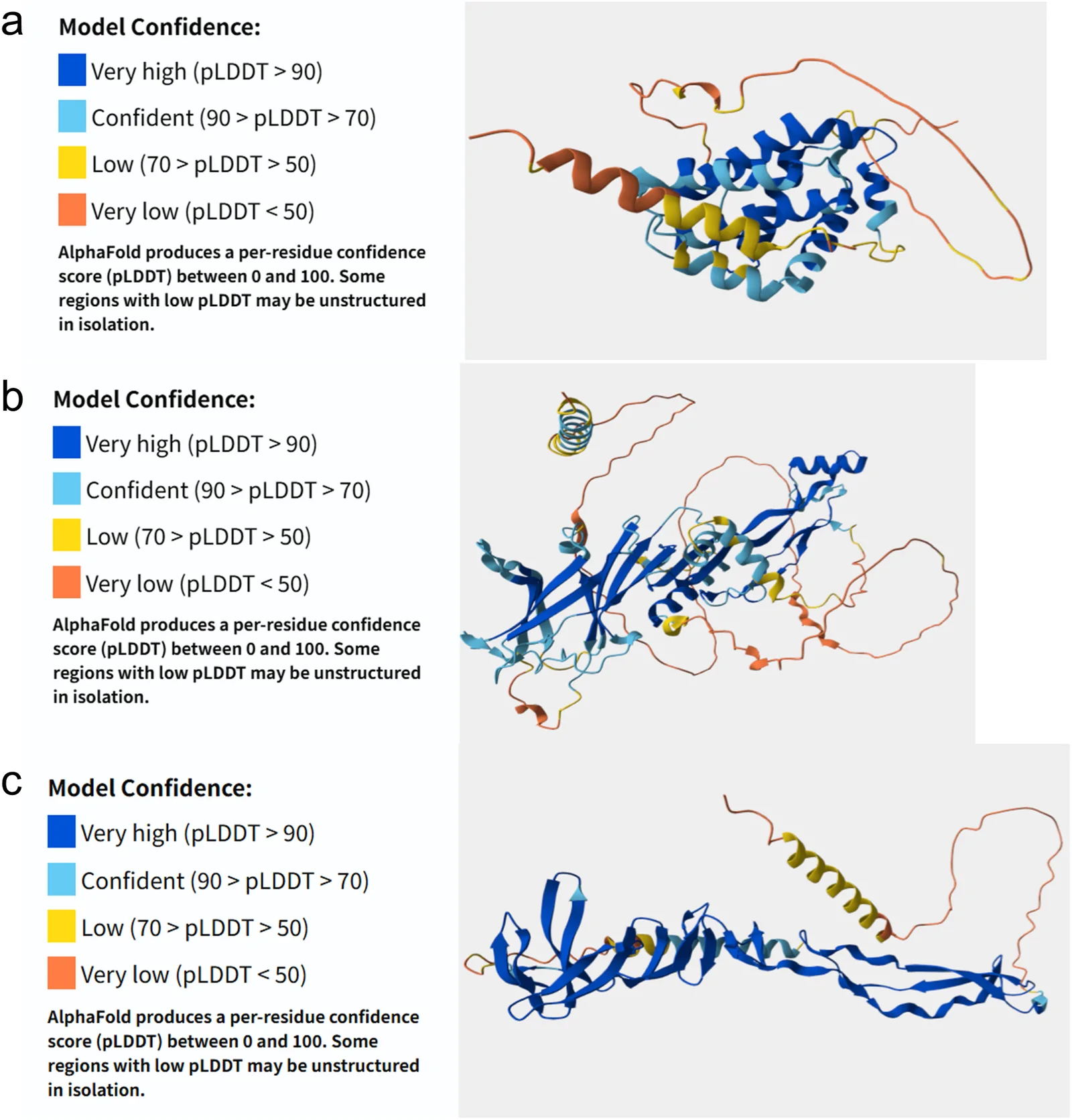
AlphaFold3 structural models and prediction confidence of representative candidate proteins. AlphaFold3-predicted structures of representative proteins carrying the selected missense substitutions. Panels show: (a) BCL2, (b) GDF5, and (c) RSPO3. Structures are colored according to per-residue pLDDT confidence scores, with blue indicating very high confidence, cyan indicating confident regions, yellow indicating low confidence, and orange indicating very low confidence. These models were used for subsequent exploratory structural assessment and molecular dynamics simulations.

### 3.11 Molecular dynamics analysis

During the 100-ns molecular dynamics simulations, the BCL2-W188G mutant showed higher RMSD than the wild-type protein during the equilibrated phase, suggesting an increased global conformational deviation from the initial structure after mutation. In parallel, both Rg and SASA were higher in BCL2-W188G than in the wild type, indicating a relatively more expanded protein conformation, reduced structural compactness, and increased solvent exposure. RMSF analysis further showed that the W188G substitution affected not only the conformational behavior around the mutation site but also increased flexibility in several distal residue regions, suggesting a redistribution of residue-level motion in BCL2. Overall, the BCL2-W188G mutant was characterized by increased global conformational deviation, reduced compactness, greater solvent exposure, and enhanced flexibility in distal regions, indicating an unfavorable effect on the conformational dynamic stability of BCL2 (Fig 7a).

**Fig 7:**
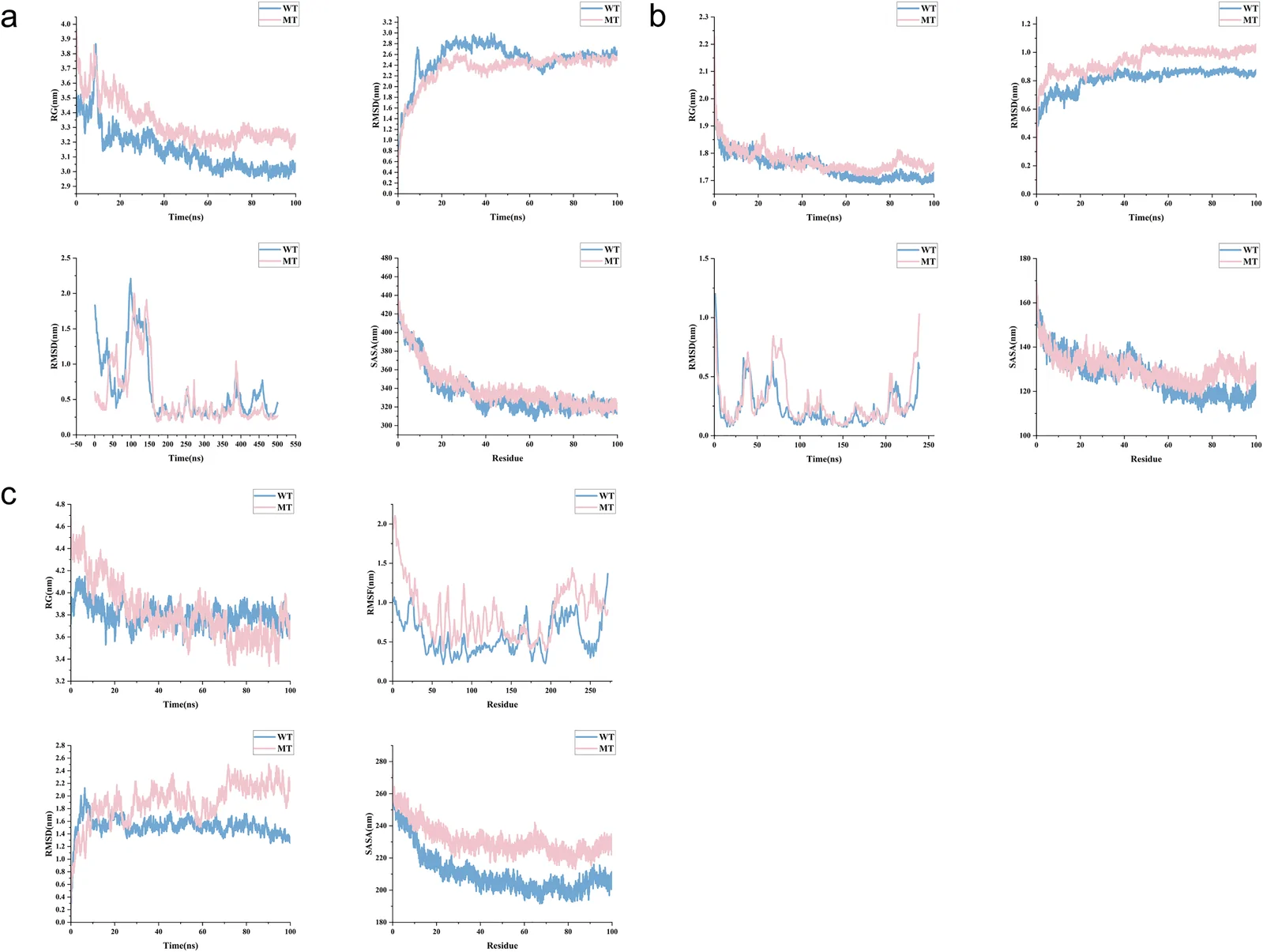
Molecular dynamics simulation analysis of representative missense variants. Molecular dynamics trajectory analysis comparing wild-type and mutant proteins during 100-ns simulations. Panels show: (a) BCL2-W188G, (b) GDF5-C400Y, and (c) RSPO3-C129Y. Curves summarize dynamic structural parameters, including root mean square deviation, root mean square fluctuation, radius of gyration, and solvent-accessible surface area. Differences between wild-type and mutant trajectories indicate mutation-related changes in conformational stability, residue flexibility, compactness, and solvent exposure.

In contrast to BCL2-W188G, the RMSD of the GDF5-C400Y mutant was not higher than that of the wild-type protein during the equilibrated phase, suggesting that the C400Y substitution did not induce a more pronounced global backbone deviation. However, Rg remained consistently higher in the GDF5-C400Y mutant than in the wild type, indicating a more expanded overall conformation and reduced structural compactness. SASA analysis showed that solvent exposure was increased in the mutant during the middle-to-late phase of the simulation, although the difference became smaller in the later phase, suggesting a relatively moderate effect of C400Y on overall solvent exposure. RMSF analysis showed that C400Y did not cause a generalized increase in protein flexibility. Instead, RMSF increased in selected distal residue regions, whereas fluctuations around the mutation site were reduced, indicating that this mutation mainly induced regional redistribution of flexibility rather than simple global conformational destabilization (Fig 7b).

The RSPO3-C129Y mutant showed more evident conformational dynamic changes. RMSD analysis showed that the C129Y mutant had markedly higher RMSD than the wild-type protein during the equilibrated phase, suggesting an increased global conformational deviation of RSPO3 after mutation. RMSF analysis further showed enhanced fluctuations around residue 129 and in multiple distal residue regions, indicating that C129Y may affect not only local structural stability but also the dynamic properties of distal residues. Rg analysis showed that the C129Y mutant was not higher than the wild type in the later phase of the simulation, suggesting that this mutation did not simply lead to global structural loosening but may instead induce conformational rearrangement or local repacking of RSPO3. SASA analysis showed persistently higher solvent-accessible surface area in the mutant than in the wild type, indicating increased surface exposure after the C129Y substitution (Fig 7c).

### 3.12 Protein ΔΔG° prediction

Protein ΔΔG° prediction showed that the three missense variants, BCL2-W188G, GDF5-C400Y, and RSPO3-C129Y, displayed varying degrees of red signals in the mutation sensitivity heatmap, suggesting that substitutions at these sites may increase the protein free energy difference. Among them, GDF5-C400Y and RSPO3-C129Y showed more pronounced red signals, indicating a potentially stronger effect on protein stability. In contrast, BCL2-W188G showed a relatively weaker red signal, suggesting a mild to moderate structural destabilizing effect (Fig 8).

**Fig 8:**
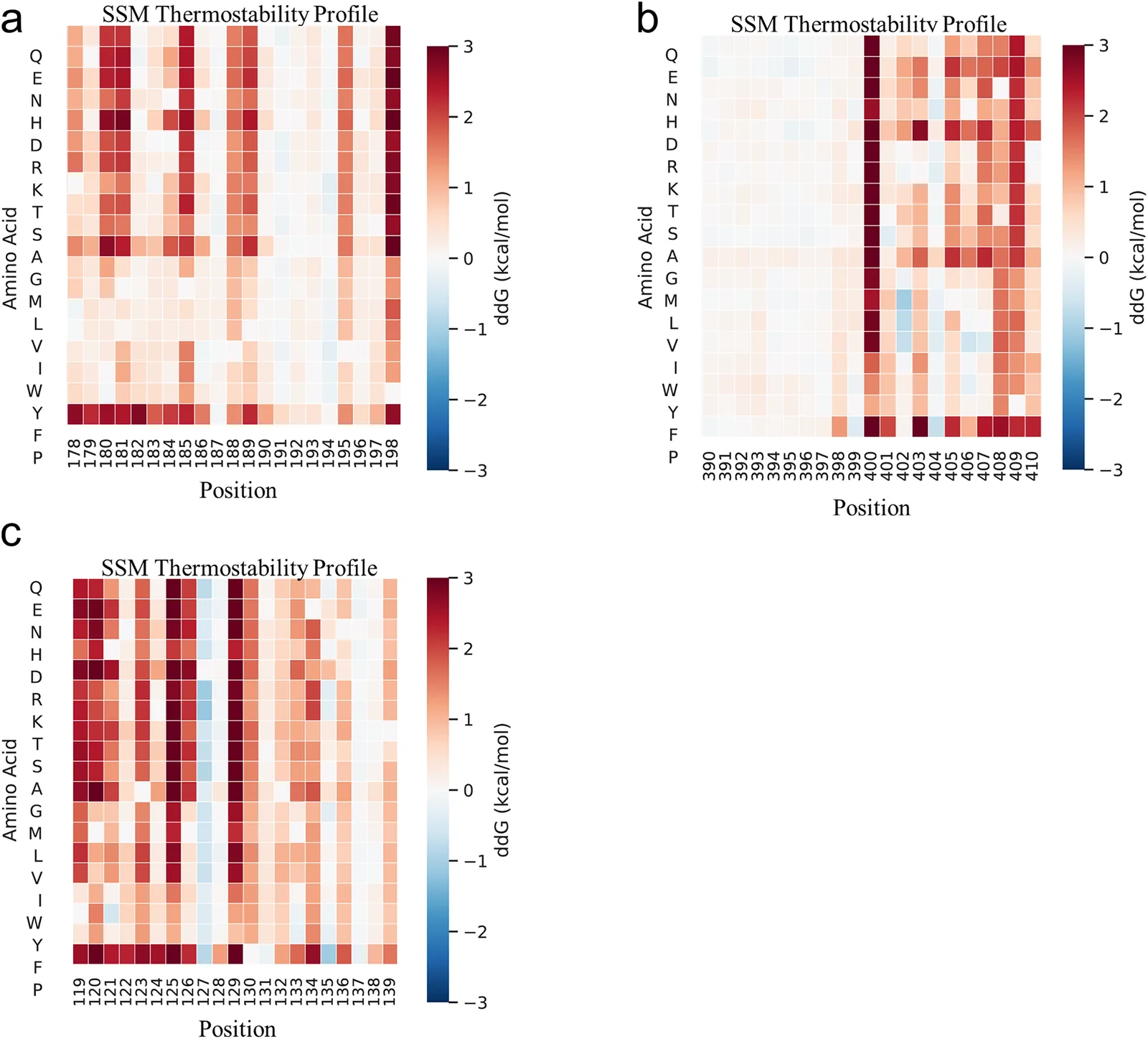
ThermoMPNN-based mutation-induced thermal stability prediction. Site-saturation mutagenesis thermostability profiles generated by ThermoMPNN for representative candidate proteins. Panels show: (a) BCL2, (b) GDF5, and (c) RSPO3. The heatmaps display predicted mutation-induced changes in free energy across residues surrounding the candidate mutation sites. Red signals indicate increased ΔΔG and a predicted destabilizing tendency, whereas blue signals indicate relatively stabilizing or less destabilizing substitutions.

## 4. Discussion

In this study, we constructed an insulin resistance-related shared metabolic genetic factor using Genomic SEM based on four metabolic phenotypes related to insulin resistance: fasting insulin, the insulin sensitivity index, triglycerides, and waist-to-hip ratio. This latent factor does not correspond to a single clinical indicator. Rather, it captures the shared genetic component extracted from multiple genetically correlated phenotypes reflecting insulin compensation, peripheral insulin sensitivity, lipid metabolism, and fat distribution. The model showed positive loadings for fasting insulin, triglycerides, and waist-to-hip ratio, and a negative loading for the insulin sensitivity index, which is consistent with the expected biological direction of insulin resistance [1,4].

These findings suggest that the genetic background of IR-related metabolic abnormalities is unlikely to be determined by a single metabolic indicator alone. Instead, it may reflect a shared genetic structure linking fat distribution, dyslipidemia, compensatory insulin secretion, and reduced insulin sensitivity. Therefore, the latent factor constructed in this study is more appropriately interpreted as an insulin resistance-related shared metabolic genetic phenotype rather than a clinical diagnostic variable for insulin resistance.

From a genetic statistical perspective, our results support a clear polygenic basis for IR-related metabolic abnormalities. The LDSC intercept was close to 1 and did not suggest substantial residual inflation attributable to confounding. Because the intercept was below 1, the corresponding negative attenuation ratio was not interpreted quantitatively. Q_SNP was used as a complementary SNP-level diagnostic and was considered together with the common-factor association when interpreting individual loci. A non-significant Q_SNP value does not by itself establish effect homogeneity.

### 4.1 Threshold-stratified GWAS results suggest that IR genetic risk involves fat distribution, lipid metabolism, and vascular metabolic regulation

In the threshold-stratified Genomic SEM analysis, we identified a set of prioritized SNPs under more stringent significance thresholds. Among these signals, rs998584 has previously been reported as a signal near the VEGFA region and has been associated with lipid-related phenotypes [36], whereas rs6905288 and rs1358980 are located in the POLR1C-related region at 6p21.1. Previous studies have suggested that rs6905288 is associated with waist-to-hip ratio, hyperlipidemia, HDL cholesterol, triglycerides, and insulin resistance-related traits [37]. Evidence for rs1358980 has mainly involved BMI-adjusted waist-to-hip ratio, waist circumference, and body fat percentage [38]. Therefore, the 6p21.1 signal observed in this study is more appropriately interpreted as a region genetically linked to fat distribution, lipid-related phenotypes, and metabolic risk. However, the causal gene, regulatory direction, and tissue context of this region remain unclear.

Because rs998584, rs6905288, and rs1358980 lack direct functional evidence showing that they influence insulin resistance through VEGFA or POLR1C, these loci should not be interpreted as established VEGFA pathway signals. Rather, they should be considered candidate regulatory regions within the shared genetic structure of IR. Further analyses integrating eQTL colocalization, chromatin interaction data, tissue-specific expression, and functional experiments are needed to clarify the effector genes and biological direction of this region.

Other prioritized signals, including rs17144687 near DTWD2, as well as rs11708173, rs16993295, rs4573145, and rs12370964, currently have relatively limited functional annotation. Their main value lies in suggesting that Genomic SEM can identify candidate regions emerging from cross-phenotype shared genetic structure that may not be readily highlighted in single-phenotype GWAS. Nevertheless, whether these loci influence IR-related metabolic states through specific genes, regulatory elements, or tissue contexts requires further validation through eQTL colocalization, chromatin interaction analysis, tissue-specific regulatory annotation, and functional experiments.

### 4.2 Fine-mapping and transcriptome integration highlight adipose tissue function, body fat distribution, and expression regulation in the IR-related latent-factor architecture

At the genetic locus level, fine-mapping further provided candidate functional regions for the signals associated with the IR-related latent factor identified by Genomic SEM. Some high-priority signals were located near PPARG, RSPO3, and SLC39A8, suggesting that shared genetic risk related to IR may involve adipose tissue function, body fat distribution, and metabolic stress regulation. PPARG is an important transcription factor involved in adipocyte differentiation and the regulation of insulin sensitivity [39]. Signals in this region suggest that lipid storage capacity and the functional state of adipocytes may contribute to genetic susceptibility to IR [10]. RSPO3 has been linked to body fat distribution and adipose progenitor cell biology, suggesting that the genetic basis of IR may depend not only on total adiposity but also on the anatomical distribution of fat deposition and the capacity for adipose depot expansion [40]. Signals near SLC39A8 further suggest that metal ion homeostasis and related metabolic stress processes may be involved in the genetic background of IR [41]. Because fine-mapping is primarily used to narrow the set of candidate causal variants, and because the single-variant association strength of some loci remains limited, these genes should be interpreted as prioritized mechanistic candidates rather than established causal genes.

Transcriptome integration analysis further supported the complexity of shared IR-related genetic signals at the level of expression regulation. In the FUSION/FOCUS results, genes such as PPARG, MLXIPL, TCF7L2, FAM13A, and CCDC92 had relatively clear metabolic interpretability. PPARG was supported by both fine-mapping and TWAS/FOCUS analyses, reinforcing the possibility that adipocyte differentiation and adipose tissue remodeling may represent important biological contexts for the shared genetic structure of IR [39]. MLXIPL is involved in glucose utilization and lipid synthesis, suggesting that abnormal glucose–lipid conversion may contribute to this genetic signal [42]. TCF7L2 is closely related to pancreatic β-cell function and insulin secretion regulation, indicating that the latent factor may reflect not only peripheral insulin sensitivity but also compensatory insulin secretion [43]. Signals for FAM13A and CCDC92 further point to fat distribution, adipocyte function, and obesity-related metabolic abnormalities [44,45].

In contrast, VEGFB and SLC22A3 showed inconsistent directions across different sCCA panels, suggesting that their effects may be tissue- or model-dependent and should therefore be interpreted cautiously. Overall, the fine-mapping and TWAS/FOCUS results were complementary in direction, suggesting that the genetic architecture captured by the IR-related latent factor may involve multiple layers, including adipose tissue function, glucose–lipid metabolic conversion, compensatory insulin secretion, and expression regulation. It should be emphasized that TWAS/FOCUS results reflect associations between genetically predicted gene expression and the latent factor and cannot, on their own, establish a causal effect of gene expression changes on IR-related phenotypes [46].

### 4.3 Enrichment analyses support adipose tissue and regulatory genomic regions as important contexts for shared IR genetic signals

Enrichment analyses further supported the importance of adipose tissue and regulatory genomic regions in shared IR genetic signals. Gene-set results showed that the candidate genes were mainly enriched for fat distribution-related traits, including waist-to-hip ratio, BMI-adjusted waist-to-hip ratio, waist circumference, hip circumference, and body shape index. They were also enriched for cardiometabolic phenotypes, including triglycerides, HDL cholesterol, fasting insulin, type 2 diabetes, metabolic syndrome, blood pressure, and coronary artery disease. This pattern is consistent with the rationale for constructing the latent factor in this study, suggesting that the factor mainly captures a shared genetic background linking fat distribution, lipid metabolism, compensatory insulin secretion, and cardiometabolic risk, rather than a single glucose- or insulin-related indicator.

Cell and tissue enrichment results showed that adipose tissue and its associated stromal or mesenchymal cell lineages may represent important contexts through which these genetic signals act. In the single-cell analysis, limb muscle mesenchymal stem cells, tracheal mesenchymal cells, and adipose-derived mesenchymal stem cells survived FDR correction. Multi-tissue expression and GTEx analyses also supported enrichment in subcutaneous and visceral adipose tissues. In parallel, active regulatory marks, including H3K4me1, H3K9ac, and H3K27ac, were significantly enriched in adipose nuclei, suggesting that some IR-related variants may be located in adipose tissue regions with enhancer activity or open chromatin features [47–49]. Partitioned LDSC analysis similarly showed that SNP heritability was mainly concentrated in conserved elements, open chromatin, enhancers, super-enhancers, transcription factor binding sites, and regions near transcription start sites. Together, these findings suggest that regulatory variants may contribute more broadly to the shared genetic structure of IR than a limited number of protein-coding variants. Chromosome-stratified analysis showed that IR-related genetic signals were unevenly distributed across the genome, with chromosomes 19, 22, 16, 12, and 4 showing relatively strong positive aggregate signals. This result provides chromosome-scale support for the polygenic nature of shared IR-related metabolic genetic susceptibility.

### 4.4 Exploratory structural analysis of candidate missense variants provides protein-level clues

Separate from the common-variant association and expression analyses, this study performed exploratory protein-level assessments of three selected ultra-rare missense substitutions. Their connection to the genetic results lies in the nomination of the corresponding candidate genes; the substitutions themselves were not implicated by the GWAS or statistical fine-mapping. Functional-impact predictions and structural modelling suitability motivated their selection and do not establish a causal link to the GWAS signals. Molecular dynamics simulations showed that BCL2-W188G, GDF5-C400Y, and RSPO3-C129Y displayed varying degrees of conformational dynamic changes.

The BCL2-W188G mutant showed higher RMSD, Rg, and SASA during the equilibrated phase, together with increased RMSF in several distal residue regions. These findings suggest that this mutation may increase global conformational deviation, reduce structural compactness, and expand solvent exposure in BCL2. Because W188G replaces a bulky aromatic tryptophan with a small glycine residue, this substitution may weaken local hydrophobic packing and spatial support, making the protein more prone to a relatively loosened conformational state. As BCL2 is involved in the regulation of apoptosis and cellular stress responses, its structural stability is important for maintaining protein interaction interfaces and regulatory function [50,51]. Therefore, the conformational loosening and distal flexibility changes induced by W188G may provide candidate structural clues for cellular homeostasis regulation under adipose inflammation, oxidative stress, or metabolic stress conditions.

In contrast, the GDF5-C400Y mutant did not show higher RMSD than the wild-type protein, suggesting that it did not induce a marked global backbone deviation. However, its persistently increased Rg, together with altered flexibility in selected distal residue regions, suggests that this mutation may mainly affect conformational compactness and regional dynamic distribution. C400Y replaces cysteine with a larger tyrosine residue, which may alter local spatial packing or cysteine-mediated structural constraints and thereby influence the dynamic stability of distal regions through long-range conformational coupling [52,53]. Given that GDF5 is involved in tissue development, cell differentiation, and tissue remodeling, and that the development of IR also involves adipose tissue remodeling, abnormal fat distribution, and altered mesenchymal cell function [54,55], the conformational expansion and flexibility redistribution associated with GDF5-C400Y may serve as a structural clue linking this candidate variant to mechanisms related to metabolic tissue remodeling.

The RSPO3-C129Y mutant showed more pronounced conformational dynamic changes. Its RMSD and SASA were higher than those of the wild-type protein during the equilibrated phase, and RMSF analysis showed enhanced fluctuations around the mutation site and in multiple distal residue regions. These findings suggest that C129Y may affect both local structural stability and distal dynamic properties. Notably, Rg was not increased in RSPO3-C129Y, indicating that this mutation does not simply cause global protein loosening but may instead induce conformational rearrangement or local repacking. RSPO3 is an important regulator of the Wnt signaling pathway and has been linked to fat distribution, adipocyte differentiation, and metabolic homeostasis [40,56]. Therefore, the increased local flexibility and solvent exposure caused by C129Y may affect the structural basis by which RSPO3 maintains its normal conformation and participates in signaling regulation, providing a structural rationale for future validation of its potential role in adipose tissue regulation and IR-related metabolic abnormalities.

It should be emphasized that these molecular dynamics results represent exploratory computational evidence and cannot directly establish causal effects of these variants on protein function, cellular behavior, or IR-related phenotypes [57]. Whether the observed conformational changes truly affect protein interactions, signal transduction, or metabolic regulation requires further validation through protein interaction assays, cellular functional experiments, and in vivo models.

#### Mutation-induced ΔΔG prediction complements molecular dynamics analysis.

This study further used ThermoMPNN to predict the ΔΔG and thermal stability effects of BCL2-W188G, GDF5-C400Y, and RSPO3-C129Y, thereby complementing the molecular dynamics-based assessment of protein dynamic stability [35]. The results showed that all three mutation sites displayed varying degrees of predicted destabilizing tendency, suggesting that these amino acid substitutions may adversely affect local or global protein structural stability. Among them, GDF5-C400Y and RSPO3-C129Y showed more pronounced predicted perturbations, suggesting that substitution of cysteine by the larger tyrosine residue may alter local spatial packing or cysteine-mediated structural constraints, thereby increasing local structural instability [58].

By comparison, the predicted thermal stability change of BCL2-W188G was relatively modest. However, because this mutation replaces a bulky aromatic tryptophan with a small glycine residue, it may still weaken local hydrophobic packing and spatial support, making the protein more prone to conformational adjustment [59,60]. When integrated with the molecular dynamics results, the predicted destabilizing tendency of GDF5-C400Y was consistent with its persistently increased Rg, suggesting that this mutation may reduce conformational compactness. The destabilizing tendency of RSPO3-C129Y was concordant with its increased RMSD, RMSF, and SASA, suggesting that it may induce conformational rearrangement, enhanced local flexibility, and increased solvent exposure. Although BCL2-W188G showed a relatively mild predicted change in thermal stability, the MD results still indicated that it may cause conformational dynamic perturbation.

Overall, ΔΔG prediction and MD analysis provided complementary evidence in the same general direction, supporting the possibility that these missense variants may affect protein structural stability to varying degrees. However, these findings remain computational predictions and require further validation through experimental structural and functional studies.

#### Limitations.

This study has several limitations. First, the input GWAS data were mainly derived from European-ancestry populations, and the generalizability of the findings to other ancestral populations requires further validation. Second, the latent factor constructed in this study reflects the shared genetic component across IR-related metabolic phenotypes. It cannot replace clinical diagnostic measures of IR and is not suitable for individual-level risk assessment. Third, novel locus identification and fine-mapping results remain influenced by the LD reference panel, regional definition, and statistical model. Therefore, candidate loci require further validation through eQTL colocalization, chromatin interaction analysis, and functional experiments. Fourth, TWAS/FOCUS can only indicate associations between genetically predicted expression and the latent factor and cannot, on its own, establish a causal role for expression changes. Finally, AlphaFold3, ThermoMPNN, and molecular dynamics simulations are all computational prediction approaches, and the 100-ns simulations can provide only exploratory structural evidence. The candidate genes and missense variants identified in this study require further validation through protein interaction assays, cellular functional experiments, and in vivo models.

## 5. Conclusion

This study used Genomic SEM to construct an insulin resistance-related latent genetic factor integrating fasting insulin, insulin sensitivity, triglycerides, and waist-to-hip ratio. The results suggest that shared IR-related genetic signals are closely linked to fat distribution, lipid metabolism, adipose tissue function, and regulatory genomic regions. Fine-mapping and TWAS/FOCUS analyses prioritized several candidate loci and genes involved in adipocyte biology, metabolic regulation, and expression control. Separate exploratory structural analyses suggested that selected ultra-rare missense substitutions may alter protein stability and conformational dynamics. These substitutions were chosen for predicted functional impact and modelling suitability rather than variant-level association or fine-mapping support. The genetic findings nominate candidates for further study, while the structural results generate substitution-specific hypotheses and do not explain or validate the common-variant GWAS associations.

## Supporting information

S1 Table GWAS summary statistics datasets included in the insulin resistance-related analysis. (DOCX)

S2 Table Pairwise genetic correlations and univariate SNP-based heritability estimates for four metabolic phenotypes. (DOCX)

S3 Table Genomic SEM common-factor model fit indices and factor parameter estimates (worksheets 3a and 3b). (XLSX)

S4 Table SNP-level associations with the insulin resistance-related latent genetic factor at P < 5 × 10^−12^ and P < 5 × 10^−16^ (worksheets 4a and 4b). (XLSX)

S5 Table Genomic risk loci identified by FUMA based on the Genomic SEM GWAS. (XLSX)

S6 Table FDR-significant genes identified by MAGMA gene-based analysis. (XLSX)

S7 Table Lead SNPs identified by FUMA functional annotation. (XLSX)

S8 Table Candidate SNPs prioritized through comparison with single-trait GWAS signals. (XLSX)

S9 Table Fine-mapping results from SuSiE and FINEMAP for major risk regions. (XLSX)

S10 Table Significant FUSION TWAS gene–tissue association signals. (XLSX)

S11 Table Overlapping FUSION TWAS signals and FOCUS credible-set results. (XLSX)

S12 Table MAGMA genes meeting the Bonferroni-corrected significance threshold. (XLSX)

S13 Table Gene-set, pathway, and phenotype ontology enrichment analyses based on Bonferroni-significant MAGMA genes. (XLSX)

S14 Table Cell-type, tissue, chromatin, and gene-expression enrichment results (worksheets 14a–14d). (XLSX)

S15 Table MendelVar Disease Ontology enrichment analysis. (XLSX)

S16 Table MendelVar Human Phenotype Ontology enrichment analysis. (XLSX)

S17 Table Chromatin annotation and partitioned heritability enrichment analysis. (XLSX)

S18 Table Chromosome-stratified summary polygenic risk score contributions. (XLSX)

S19 Table Functional annotations of the three missense variants selected for exploratory structural analysis. (DOCX)

S1 File Exploratory single-factor model object for the insulin resistance-related metabolic phenotypes (RData format). (RDATA)
