# Supplementary material for: Integrative genomic SEM analysis of shared genetic architecture and candidate genes for insulin resistance–related metabolic traits
Source: PLoS One. 2026 Oct 29;21(10):e0361601. doi: 10.1371/journal.pone.0361601 (PMC13719024; doi:10.1371/journal.pone.0361601)
Supplement: Supplementary file 1 [file pone.0361601.s001.docx]

Supplementary Table 1 GWAS summary statistics datasets included in the insulin resistance-related analysis

| Trait | Sample size (n) | Study population | DOI |
| --- | --- | --- | --- |
| Fasting insulin | 51750 | European ancestry | 10.1038/ng.2274 |
| Insulin sensitivity index | 53657 | European ancestry | 10.1038/s41588-023-01408-9 |
| Triglycerides | 115082 | European ancestry | 10.1371/journal.pbio.3001547 |
| Waist-to-hip ratio | 342784 | European ancestry | 10.1038/s41588-024-02035-8 |

Abbreviations: FI, fasting insulin; ISI, insulin sensitivity index; TG, triglycerides; WHR, waist-to-hip ratio; IR, insulin resistance; GWAS, genome-wide association study; SEM, structural equation modeling.

Note: The table summarizes the GWAS summary statistics datasets used to construct the IR-related latent genetic factor. All datasets were derived from individuals of European ancestry.
