# Supplementary material for: Integrative genomic SEM analysis of shared genetic architecture and candidate genes for insulin resistance–related metabolic traits
Source: PLoS One. 2026 Oct 29;21(10):e0361601. doi: 10.1371/journal.pone.0361601 (PMC13719024; doi:10.1371/journal.pone.0361601)
Supplement: Supplementary file 2 [file pone.0361601.s002.docx]

Supplementary Table 2. Pairwise genetic correlations and univariate SNP-based heritability estimates for the four IR-related metabolic phenotypes

| Phenotype | FI | ISI | TG | WHR |
| --- | --- | --- | --- | --- |
| FI | 0.0815 (0.0121) | −0.8392 (0.0847) | 0.3654 (0.1083) | 0.4571 (0.0695) |
| ISI | −0.8392 (0.0847) | 0.1042 (0.0117) | −0.3319 (0.0571) | −0.4424 (0.0536) |
| TG | 0.3654 (0.1083) | −0.3319 (0.0571) | 0.1478 (0.0160) | 0.3469 (0.0516) |
| WHR | 0.4571 (0.0695) | −0.4424 (0.0536) | 0.3469 (0.0516) | 0.1352 (0.0084) |

**Abbreviations:** FI, fasting insulin; ISI, insulin sensitivity index; TG, triglycerides; WHR, waist-to-hip ratio; IR, insulin resistance; SNP, single-nucleotide polymorphism.

**Note:** Off-diagonal entries represent pairwise genetic correlation estimates between traits, with corresponding standard errors shown in parentheses. Diagonal entries represent univariate SNP-based heritability estimates for each trait. The negative genetic correlations between ISI and FI, TG, and WHR are consistent with the inverse relationship between insulin sensitivity and insulin resistance-related metabolic traits.
