# Supplementary material for: Integrative genomic SEM analysis of shared genetic architecture and candidate genes for insulin resistance–related metabolic traits
Source: PLoS One. 2026 Oct 29;21(10):e0361601. doi: 10.1371/journal.pone.0361601 (PMC13719024; doi:10.1371/journal.pone.0361601)
Supplement: Supplementary file 3 [file pone.0361601.s019.docx]

Supplementary Table
Functional annotations of the three missense variants selected for exploratory structural analysis

| **Annotation** | **BCL2** | **GDF5** | **RSPO3** |
| --- | --- | --- | --- |
| Variant ID | rs2144321090 | rs74315387 | rs1352673048 |
| Protein change | p.Trp188Gly | p.Cys400Tyr | p.Cys129Tyr |
| CADD | 32 likely deleterious | 31 likely deleterious | 32 likely deleterious |
| REVEL | 0.756 likely disease causing | 0.986 likely disease causing | 0.816 likely disease causing |
| SIFT | 0 deleterious | 0 deleterious | 0 deleterious |
| PolyPhen | NR | 1 probably damaging | 0.996 / 0.984 probably damaging |
| MetaLR | 0.194 tolerated | 0.691 damaging | 0.9 damaging |
| Mutation Assessor | 0.911 medium | NR | 0.991 high |
| Clinical significance | NR | Likely pathogenic / pathogenic | NR |
| Transcript IDs | ENST00000333681.5 ENST00000398117.1 | ENST00000374369.8 ENST00000374372.1 | ENST00000356698.9 ENST00000368317.3 |
| gnomAD AF (v4.1.1) | Not detected | Not detected | 6.203 × 10⁻⁷ AC = 1; AN = 1,612,204 |

Functional annotations were obtained from the Ensembl exports. Scores and prediction categories are reproduced as reported; NR denotes an unavailable annotation, not a benign classification. The displayed Mutation Assessor values should be interpreted with their reported categories rather than as probabilities of pathogenicity.

Both transcript records are listed for each variant. RSPO3 PolyPhen scores of 0.996 and 0.984 correspond to ENST00000356698.9 and ENST00000368317.3, respectively. The other displayed predictions agree between the two records for each variant.

The GDF5 clinical classifications are reproduced from the Ensembl clinical-significance field. They do not establish pathogenicity for insulin resistance. The exports do not specify the associated conditions or review status.

AF, allele frequency; AC, allele count; AN, allele number. gnomAD queries used GRCh38 and the specific alternate allele. The RSPO3 AF is the combined exome/genome value. Not detected denotes a browser result of “Variant not found”; a numerical AF of zero was not assigned.

Functional annotations support the choice of these variants for exploratory analysis within nominated candidate genes; selection also considered structural-modeling suitability. BCL2 predictions were mixed, including a tolerated MetaLR classification. The three variants were neither GWAS lead variants nor fine-mapped causal variants. These annotations do not establish a causal link to the common-variant GWAS signals.
